# Crayfish Impact Desert River Ecosystem Function and Litter-Dwelling Invertebrate Communities through Association with Novel Detrital Resources

**DOI:** 10.1371/journal.pone.0063274

**Published:** 2013-05-07

**Authors:** Eric K. Moody, John L. Sabo

**Affiliations:** Arizona State University School of Life Sciences, Tempe, Arizona, United States of America; Institute of Botany, Czech Academy of Sciences, Czech Republic

## Abstract

Shifts in plant species distributions due to global change are increasing the availability of novel resources in a variety of ecosystems worldwide. In semiarid riparian areas, hydric pioneer tree species are being replaced by drought-tolerant plant species as water availability decreases. Additionally, introduced omnivorous crayfish, which feed upon primary producers, allochthonous detritus, and benthic invertebrates, can impact communities at multiple levels through both direct and indirect effects mediated by drought-tolerant plants. We tested the impact of both virile crayfish (*Orconectes virilis*) and litter type on benthic invertebrates and the effect of crayfish on detrital resources across a gradient of riparian vegetation drought-tolerance using field cages with leaf litter bags in the San Pedro River in Southeastern Arizona. Virile crayfish increased breakdown rate of novel drought-tolerant saltcedar (*Tamarix ramosissima*), but did not impact breakdown of drought-tolerant seepwillow (*Baccharis salicifolia*) or hydric Fremont cottonwood (*Populus fremontii*) and Gooding's willow (*Salix goodingii*). Effects on invertebrate diversity were observed at the litter bag scale, but no effects were found at the cage scale. Crayfish decreased alpha diversity of colonizing macroinvertebrates, but did not affect beta diversity. In contrast, the drought-tolerant litter treatment decreased beta diversity relative to hydric litter. As drought-tolerant species become more abundant in riparian zones, their litter will become a larger component of the organic matter budget of desert streams which may serve to homogenize the litter-dwelling community and support elevated populations of virile crayfish. Through impacts at multiple trophic levels, crayfish have a significant effect on desert stream ecosystems.

## Introduction

Ecosystems around the world are threatened by anthropogenic global change, particularly the spread of introduced species [1]. Humans both directly spread species into previously unoccupied areas and also indirectly cause species range expansions and colonization of novel habitats through global change [2]. In most ecological communities there are now multiple, interacting sources of novel species that form what have been called novel ecosystems [3]. Further, the course of novel ecosystem dynamics may be determined not just by the direct effects of all novel species in the system, but also by interactions between different novel species.

As ecosystems face multiple disturbances, surprising ecological consequences are more likely to occur [4]. Arid and semiarid streams are impacted by introduced aquatic and riparian species as well as declining precipitation and water tables [5], [6], [7]. Flow regime is an extremely important driver of community structure in arid and semiarid streams, and streams are more heavily impacted by flow alterations in arid regions than their temperate counterparts [8], [9], [10]. Water availability in some desert streams has already become increasingly variable over the past century [7], and this variability has severe consequences for lotic organisms which are adapted to living under their native flow regimes [11]. Sabo and Post identified low flow anomalies as an underappreciated source of catastrophic temporal environmental variation in an analysis of 105 streams throughout the United States [12]. Changes to flow regime can alter macroconsumer effects on stream ecosystem function, competition between native and introduced species, as well as macroinvertebrate community composition [13], [14], [15], [16]. Additionally, altered flow regimes can have indirect effects on aquatic benthic communities through effects on riparian flora.

Due to changes in flood intensity, base flows, and groundwater depth, riparian vegetation communities shift from hydric species to mesic drought-tolerant species as variation in water availability increases [17], [18]. These drought-induced community shifts have well-studied effects on riparian systems but little-known impacts on stream ecosystems [18]. Allochthonous detrital inputs can form the base of the food web in some stream systems and represent an important flow of energy between aquatic and riparian systems [19], [20]. Global change-induced shifts in riparian vegetation communities and their resulting effects on detrital inputs impact aquatic ecosystems worldwide [21]. We are considering drought-tolerant plant litter, whether from native or introduced species, to be a novel resource in desert streams because it was historically not likely to be a large component of perennial desert stream detrital pools [17], [18]. Introduction of novel litter from drought-tolerant plants is likely to impact benthic organisms because leaves of hydric and drought-tolerant plants differ in quality and structure [22], [23], [24], [25]. In semiarid riparian zones both native and introduced species establish populations along stream reaches with altered flow regimes [18], providing a mixture of novel and historically present organic matter sources for detritivores.

While invasive or native status of detrital resources does not explain differences in litter breakdown per se, differences in stoichiometry between native and invasive plants can account for differences in decomposition (e.g., [26]). Food quality, as defined by the elemental ratios of key nutrients such as C∶N, is a highly important factor in determining detritivore foraging [27], [28]. Introduced, drought-tolerant saltcedar (*Tamarix ramosissima*) leaves have been found to be of higher quality than cottonwood (*Populus)* and willow (*Salix*) leaves, thus saltcedar may be a preferred resource of stream detritivores [24], [25], [29]. While North American desert streams often lack obligate detritivores (e.g., [30]), many of these streams have been invaded by omnivorous crayfish which may consume these detrital resources.

Many species of crayfish consume autochthonous and allochthonous resources, and through competition, predation, and ecosystem engineering they may have both direct and indirect effects on invertebrates [31], [32], [33], [34], [35]. Introduced crayfish are likely to have pronounced effects in systems such as the Colorado River basin where there were no native omnivorous analogs [36]. While Colorado River basin streams have historically hosted native omnivorous fishes such as longfin dace (*Agosia chrysogaster*) and desert sucker (*Catostomus clarki*), these fishes do not feed on coarse particulate detritus [37].

Omnivorous macroconsumers can be highly important in the breakdown of leaf litter in a diverse array of aquatic systems, yet their role as detritivores has not been examined in North American desert streams. Selective feeding by crayfish can have both direct and indirect effects on benthic communities, and in some streams the indirect effects of herbivores and detritivores are greater than the direct effects of predators in shaping benthic community composition [31], [38]. If crayfish feed selectively on certain species of riparian detritus as well as benthic invertebrates, they can impose both direct and indirect impacts on desert river benthos. This effect may be of special interest if crayfish disproportionately affect availability of introduced saltcedar detritus as they may form a novel pathway in the food web. Several examples of novel consumers relying on novel resources have been reported (e.g., [39], [40]), thus we investigated whether such a situation may occur in desert streams.

In this paper we ask how a combination of novel riparian vegetation (an allochthonous resource to the food web) and novel omnivores alter community structure and ecosystem function in a desert river. Our overarching hypothesis is that omnivores (the crayfish *Orconectes virilis*) alter community structure primarily by hastening decomposition of high-quality novel litter inputs. We test two specific hypotheses. First, that crayfish increase leaf litter decomposition by efficient shredding of allochthonous plant resources. We predict that decomposition of all litter species will be faster in the presence of crayfish, but in the San Pedro River in Southeast Arizona this effect will be strongest for saltcedar due to evidence of its high food quality and its novelty in the system. Second, we hypothesize that litter-dwelling invertebrate diversity will decrease in response to crayfish presence via direct predation and changes in resource availability caused by crayfish feeding. We predict that both alpha and beta diversity of litter-dwelling invertebrates will be lower in the presence of crayfish.

## Methods

### Ethics Statement

We did not require ethical approval to conduct this study as we did not handle or collect samples of any vertebrates. We received permission from a private landowner (Sandy Anderson) and the Bureau of Land Management (Permit #4180 (AZ421)) to access study sites. Crayfish were sampled under an Arizona fishing license held by EKM (#HM145846).

### Study Sites

**Figure 1:**
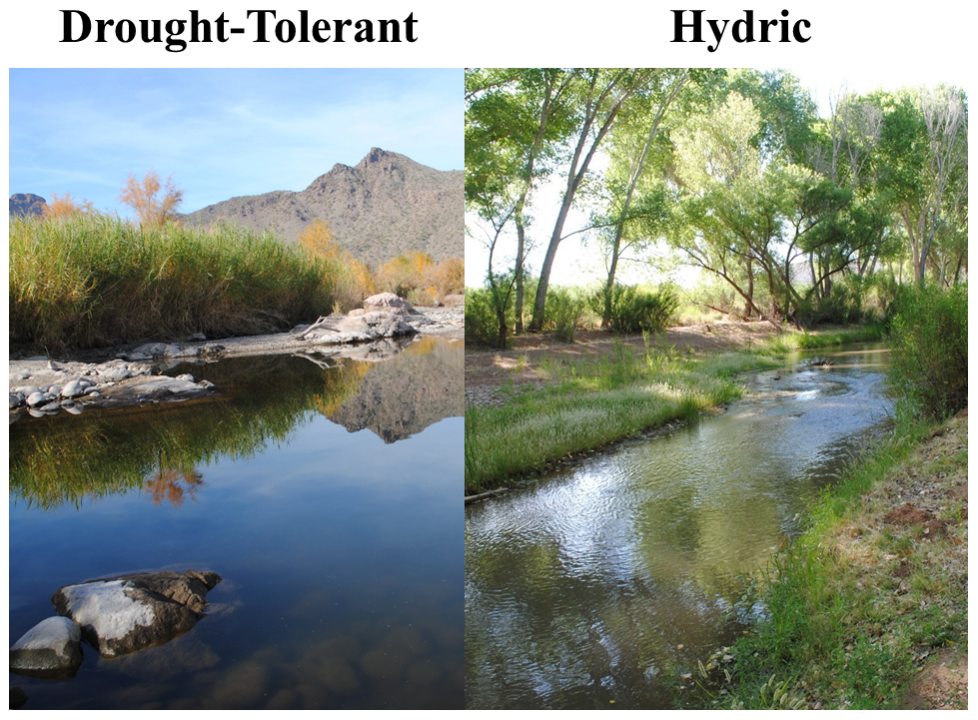
A drought-tolerant riparian plant community dominated by saltcedar (*Tamarix ramosissima*) and seepwillow (*Baccharis salicifolia*) at the Salt River (Maricopa Co., AZ) and a hydric riparian plant community dominated by Fremont cottonwood (*Populus fremontii*) and Gooding's willow (*Salix goodingii*) at the San Pedro River (Cochise Co., AZ). Photos taken by EKM.

This research was conducted in the San Pedro River, a semiarid river in the Colorado River basin draining northeastern Sonora in Mexico and southeastern Arizona in the United States. Similar to many rivers draining arid and semiarid catchments, the San Pedro is spatially and temporally intermittent, with alternating perennial and intermittent reaches [41]. This study incorporated one perennial reach, Grayhawk Nature Center (hereafter Grayhawk) (31.604°N, 110.153°W), and one reach that is intermittent in very dry years, Charleston (31.630°N, 110.178°W). We chose to conduct this study at these two sites to capture a range of environmental conditions present in the San Pedro. Neither reach dried completely during the study period, but Charleston (mean±SE: 23.5°C±0.29) was warmer than Grayhawk (mean±SE: 23.0°C±0.33) throughout the study. Riparian vegetation at the study sites is dominated by Fremont cottonwood (*Populus fremontii*), Gooding's willow (*Salix goodingii*), seepwillow (*Baccharis salicifolia*), and saltcedar. These species can be classified along a gradient of drought tolerance [42], with declining streamflows and groundwater levels causing shifts to drought-tolerant species such as saltcedar [18]. Extremes of this gradient are dominated by a drought-tolerant community or hydric community (Figure 1), although we emphasize that all of these species often co-occur along many desert rivers. The river is inhabited by non-native virile crayfish and red swamp crawfish (*Procambarus clarkii*), but virile crayfish numerically dominate the study reaches [43]. Introduced fish present at our study sites include mosquitofish (*Gambusia affinis*), black bullhead catfish (*Ameiurus melas*), channel catfish (*Ictalurus punctatus*), green sunfish (*Lepomis cyanellus*), largemouth bass (*Micropterus salmoides*), and common carp (*Cyprinus carpio*) in addition to the native longfin dace and desert suckers. Additionally, the river hosts a diverse benthic invertebrate community of insects, crustaceans, and gastropods.

### Experimental Design

We deployed sixteen cages in a generalized randomized block design at each reach during the dry season on May 24, 2011 and removed them immediately preceding the first monsoonal flood on June 24, 2011. The experiment was conducted during the warm, dry season because crayfish are active in processing leaf litter during warmer months [44] and because arid riparian plants often drop leaves in response to water stress during the dry season [45]. Although this is a relatively short time scale, this period reflects the time when crayfish are actively feeding on leaf litter before it is exported downstream in monsoonal floods [23]. Cages measured approximately 0.2 m^2^ in area and were covered with 8 mm^2^ mesh on the upstream and downstream ends as well as 48 mm^2^ mesh above the water to prevent interference from birds and mammals. This mesh size excluded movement by large crayfish and most fish but allowed passage by small fish such as mosquitofish and most other invertebrates including young-of-year (YOY) virile crayfish, which were present at Charleston but not Grayhawk. Cages were filled with natural periphyton-covered stream sediments and set in the stream for forty-eight hours to settle before we added treatments.

Each cage received one level of a virile crayfish treatment and one level of a leaf litter treatment. Crayfish treatment levels consisted of either a control of no crayfish or one (mean initial carapace length = 21 mm) virile crayfish representing a conservative density of 5/m^2^, somewhat less than published densities in a different Colorado River basin stream [36]. Leaf litter treatments consisted of litter bags (pecan bags; Gulf Coast Bag and Bagging Co., Houston, TX) containing 3.5 g of either hydric species (Fremont cottonwood and Gooding's willow) or more drought-tolerant species (saltcedar and seepwillow). Each litter bag consisted of a single species, with separate bags of each species per treatment level in all cages receiving that level. We chose to deploy litter bags in this way because the species-pairs chosen represent contrasting communities that dominate perennial and intermittent reaches of desert rivers, replicating litter conditions experienced by stream detritivores across these differing hydrologic regimes. As there are generally non-additive effects of litter species mixing [46], it is important to consider these species-pairs together to capture dynamics that occur at a broader scale in the river.

Senescent leaves of all species were collected from the study reaches of the San Pedro in 2010, except saltcedar which was collected from the Salt River above Granite Reef Dam. Litter bags of cottonwood and saltcedar were retrieved weekly, while bags of willow and seepwillow were retrieved biweekly. This arrangement provided differing initial standing stocks of litter for each species per treatment which reflected general patterns of abundance of these riparian species at perennial and intermittent sites along streams in Arizona [18]. Breakdown rate (*k*) was calculated for each species/crayfish treatment combination following [47]. All invertebrates were rinsed from leaf litter bags before processing and identified to genus except physid snails and chironomid midge larvae; the latter were identified to subfamily. Additionally, each cage was sampled for invertebrates on the final date by sweeping a d-frame net for 10 seconds after removal of litter bags and crayfish. Virile crayfish were measured and weighed at the beginning and end of the experimental period. All crayfish were held for a 24-hour period with no food before being weighed each time to ensure that gut contents did not factor into weight measurements. Water temperature was measured every thirty minutes from June 10 through June 24 at both sites with a HOBO Water Temp Pro v2 temperature logger (Onset Computer Corporation, Pocasset, MA).

### Statistical Analysis

We performed two primary sets of analyses. To test our first hypothesis, we assessed whether litter treatment or crayfish treatment impacted leaf litter breakdown rate and whether crayfish growth differed across litter treatments. To test our second hypothesis, we evaluated whether invertebrate community composition and diversity differed across treatments and examined the magnitude and significance of direct and indirect effects of crayfish presence.

In all analyses except multivariate analyses we have treated site as a random effect to control for variation between sites. We tested assumptions of normality and equal variance of residuals of all models using Shapiro-Wilk and Levene's test, respectively. We tested differences in virile crayfish growth and log-transformed breakdown rate of leaf litter species using a generalized mixed effects model with site treated as a random effect. We performed Tukey-Kramer post-hoc tests to test specific comparisons.

We tested differences in the invertebrate community (as density per gram ash-free dry mass (AFDM) leaf litter in leaf bag samples and per m^2^ in cage samples) across treatments and sites using a non-metric multidimensional scaling (NMDS) ordination with zero-adjusted Bray-Curtis distance matrices [48], [49]. NMDS was run with up to twenty random starts, with a maximum acceptable stress proportion of 0.3. We excluded several invertebrate taxa that were present in very low abundance (<1% of individuals sampled) from NMDS analysis or grouped them together at the family level to reduce the influence of rare species [49]. To avoid violations of independence, NMDS was performed only on data from the fourth and final week of the experiment and litter bag samples from both litter types per cage were pooled. We calculated linear correlation coefficients between density of each taxon and the NMDS axes to relate individual taxa to factors that influenced overall community composition [16], [49]. While this approach assumes linear relationships between density and the NMDS axes which may not be entirely realistic, it provides a rough approximation of the relationships between taxa and the factors controlling the ordination. We tested whether alpha diversity (measured by the Shannon-Wiener index) differed between treatments and sites using ANOVA and whether beta diversity (measured as the slope of the species-area curve (*sensu* [50])) of invertebrates colonizing leaf litter varied between treatments and sites using multivariate ANOVA (MANOVA) with 4999 permutations (using the *adonis* command in the vegan package of R) as this analysis is more robust than ANOSIM [51]. We treated site as a random effect in both models.

We used a partial least squares (PLS) path model to test contribution of direct and indirect effects of virile crayfish on invertebrate colonization [52]. We first used binomial generalized linear models (GLMs) to test whether crayfish had any effect on invertebrate abundance, density, and alpha diversity from litter bags and cages from the final week of the experiment. Alpha diversity was measured at the lowest identifiable taxonomic level for each group, and all groups were included. While the Shannon-Wiener index is subject to bias in small sample sizes because of potentially incomplete representation of the community [53], our methods were designed to sample nearly the entire community that was residing within leaf bags and cages. Therefore, we have chosen to use the Shannon-Wiener diversity index as opposed to any of the corrected indices for small sample sizes (e.g., [53]). We conducted path analyses to determine whether leaf litter breakdown rate mediated any crayfish effects that were found to be significant in the GLMs. 95% confidence intervals of path coefficients were estimated with bootstrapping with 100 resamples. We did not conduct path analyses for the whole cage invertebrate samples because crayfish did not affect any invertebrate response variables at the cage level. We performed all statistical analyses with the statistical software R version 2.14 using the car [54], lme4 [55], plspm [56], sfsmisc [57], and vegan [51] packages. All data will be made available upon request to the corresponding author.

## Results

### Leaf Litter Breakdown

**Figure 2:**
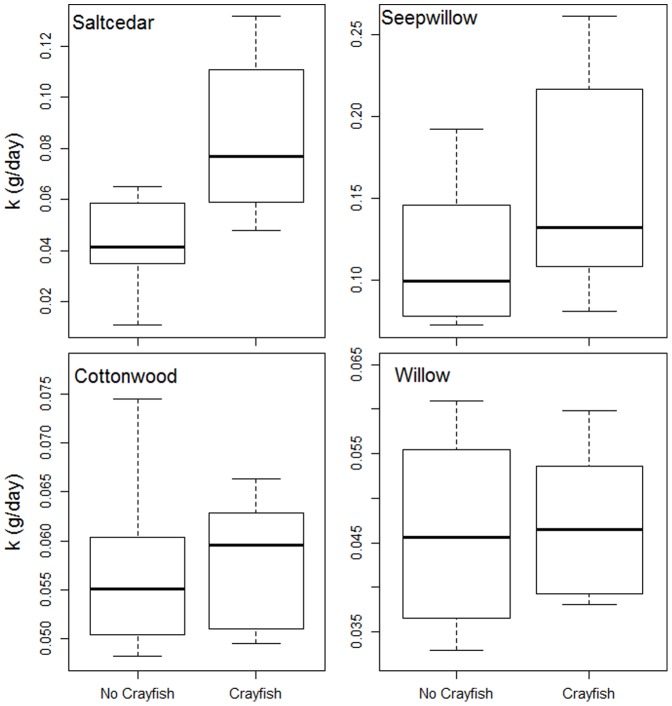
Effect of virile crayfish presence on breakdown rate (k) of four species of leaf litter. Crayfish significantly increased breakdown rate of saltcedar (Tukey-Kramer post-hoc test, p<0.01), but did not impact breakdown of seepwillow (Tukey-Kramer post-hoc test, p = 0.59), cottonwood (Tukey-Kramer post-hoc test, p = 1.00), or willow (Tukey-Kramer post-hoc test p = 1.00).

Leaf litter breakdown rate differed among leaf species (ANOVA, *F* = 31.7, *df* = 3), crayfish presence (ANOVA: *F* = 13.9, *df* = 1), and the interaction between species and crayfish presence (ANOVA: *F* = 5.1, *df* = 3; Table 1). Results of mixed-effects models do not include p-values due to uncertainty in residual degrees of freedom. Seepwillow leaves decomposed faster than cottonwood, willow, and saltcedar (Tukey-Kramer post-hoc test: *p*<0.01 for all three), but there were no significant differences between the other three species (Table 1). Breakdown rates of all species were higher at the warmer, occasionally intermittent Charleston site than at Grayhawk (Tukey-Kramer post-hoc test: *p*<0.01). Virile crayfish presence did have a significant effect on breakdown rates across species (Tukey-Kramer post-hoc test: *p*<0.01); however, direct comparisons revealed that virile crayfish significantly increased the breakdown rate only of saltcedar (Tukey-Kramer post-hoc test: *p*<0.01; Figure 2). Crayfish caused saltcedar breakdown rate to differ from willow (Tukey-Kramer post-hoc test: *p* = 0.04), but not from seepwillow (Tukey-Kramer post-hoc test: *p* = 0.07) or cottonwood (Tukey-Kramer post-hoc test: *p* = 0.42; Table 1). Growth of virile crayfish in cages did not differ significantly across leaf litter treatments (Tukey-Kramer post-hoc test: *p* = 0.148).

**Table 1:** ANOVA table and Tukey's post-hoc test results for breakdown rate of leaf litter by species and virile crayfish presence.

Factor	Df	SS	MS	F	p
Leaf Species	3	9.906	3.302	31.674	
Saltcedar-Cottonwood					0.999
Saltcedar-Willow					0.315
Seepwillow-Cottonwood					**<0.001**
Seepwillow-Willow					**<0.001**
Seepwillow-Saltcedar					**<0.001**
Willow-Cottonwood					0.378
Crayfish Presence	1	1.446	1.446	13.874	
Crayfish-No Crayfish					**0.003**
Leaf Species*Crayfish	3	1.605	0.535	5.131	

P values are presented only for Tukey's post-hoc tests due to uncertainty in calculating the denominator degrees of freedom in mixed models.

### Invertebrate Community

**Figure 3:**
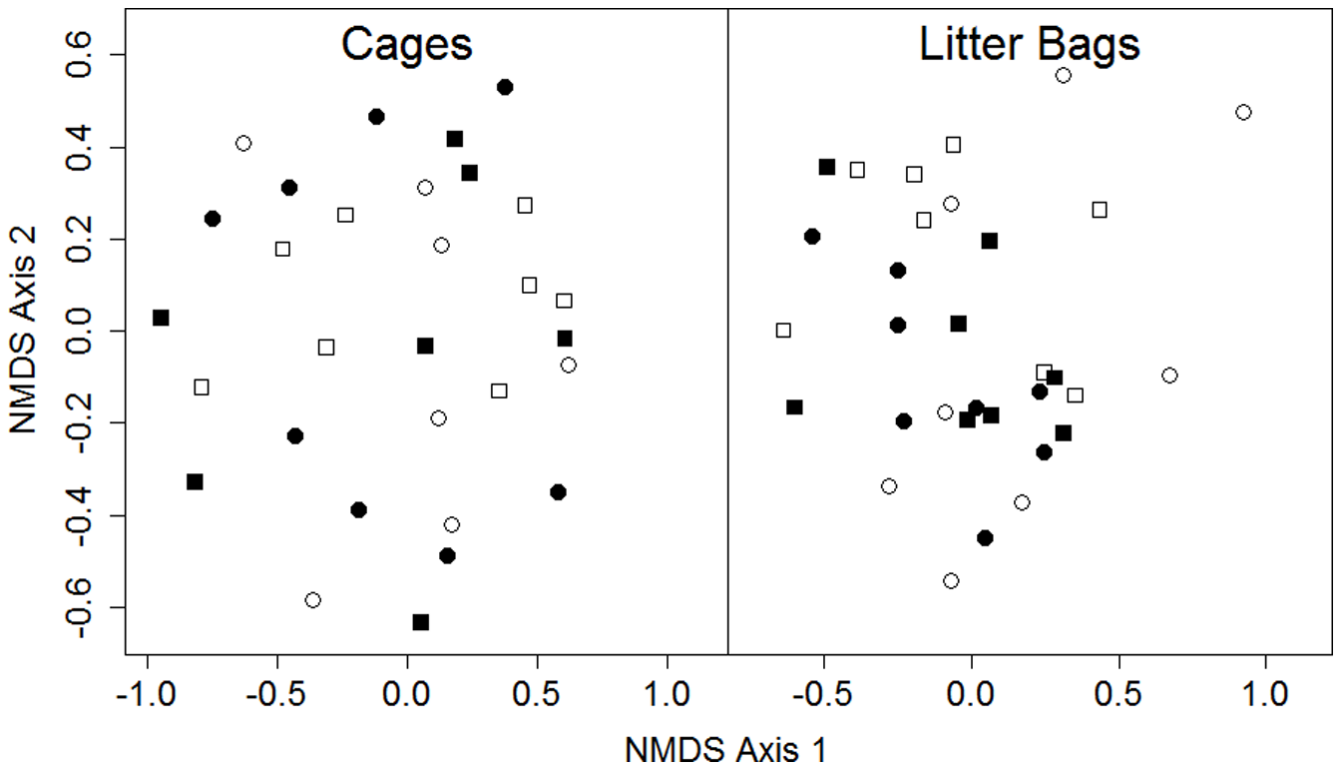
Nonmetric Multidimensional Scaling (NMDS) plot of invertebrate density from cages (Stress = 0.190) and leaf litter bags (Stress = 0.208) on the final day of the experiment. Circles represent cages with crayfish, squares represent cages without. Open symbols represent drought-tolerant litter cages, closed symbols represent hydric litter cages.

Our measurements of alpha diversity included sixteen taxa at the litter bag scale and nineteen at the cage scale (Table 2). Neither alpha nor beta diversity of invertebrate communities differed across experimental treatments at the cage level; hence we focus on the invertebrates colonizing litter bags for the remainder of this section (Table 3). Alpha diversity of litter bag invertebrates was significantly lower in the crayfish treatment (mean±SE: 0.68±0.08) than the crayfish-free treatment (mean±SE: 1.01±0.11) (ANOVA: *F* = 6.145, *df* = 1,29, *p = *0.02), but the litter treatment had no effect (ANOVA: *F* = 0.311, *df* = 1,29, *p* = 0.58). In contrast, virile crayfish presence was not a significant predictor of beta diversity of litter bag invertebrates (MANOVA: *F* = 1.3, *df* = 1,29, *p* = 0.30), but the litter treatment did have a significant effect (MANOVA: *F* = 2.6, *df* = 1,29, *p* = 0.04) with the drought-tolerant litter being associated with reduced beta diversity (Figure 3, Table 3).The interaction term was not significant in any of the models of invertebrate diversity (Table 3). While litter-dwelling invertebrate beta diversity differed between litter treatments, there was no distinct community separation between the treatments in the NMDS ordination (Figure 3). Examining trends in particular taxa reveals taxon-specific responses to changes in litter and virile crayfish presence (Table 4). The mayfly *Leptohyphes* (*r* = 0.63), physid snails (*r* = 0.53), *Tabanus* larvae (*r* = 0.32), and coenagrionid damselfly naiads (*r* = 0.32) all exhibited strong positive correlations with NMDS Axis 2 (Table 4). Non-predatory midge larvae (*r* = −0.47), predatory midge larvae (*r* = −0.42), the amphipod *Hyalella* (*r* = −0.41), and physid snails (*r* = −0.33) exhibited strong negative correlations with NMDS Axis 1 (Table 4).

**Table 2:** Benthic macroinvertebrate taxon richness in cages and litter bags from the fourth week of the experiment.

	*Cages*			*Litter Bags*		
Treatment	Crayfish	No Crayfish	Total	Crayfish	No Crayfish	Total
Drought-Tolerant Litter	11	16	18	8	9	11
Hydric Litter	13	13	14	10	9	11
Total	14	18	19	12	12	16

Taxa were defined at the lowest identifiable level, which was genus or species except for Chironomidae (split into Tanypodinae and non-Tanypodinae).

**Table 3:** ANOVA table for alpha diversity (Shannon-Wiener Index) and multivariate ANOVA for beta diversity (slope of the species-area relationship) of arthropod communities colonizing cages and leaf litter bags on the final week of incubation.

	*Cages*					*Litter Bags*				
Predictor	Df	SS	MS	F	p	Df	SS	MS	F	p
*α-Diversity*										
Crayfish	1	0.325	0.325	0.887	0.354	1	0.875	0.875	6.145	**0.019**
Litter	1	0.004	0.004	0.01	0.919	1	0.044	0.044	0.311	0.582
Crayfish*Litter	1	0.152	0.152	0.414	0.525	1	0.152	0.152	1.068	0.310
Residual	28	10.273	0.367			28	3.978	0.142		
*β-Diversity*										
Crayfish	1	0.175	0.175	1.076	0.379	1	0.134	0.134	1.336	0.289
Litter	1	0.005	0.005	0.031	0.969	1	0.278	0.278	2.769	**0.031**
Crayfish*Litter	1	0.022	0.022	0.132	0.940	1	0.233	0.233	2.319	0.069
Residual	28	4.567	0.163			28	2.809	0.100		

ANOVA was run over 4999 permutations.

**Table 4:** Pearson's correlation coefficients (r) between density of taxa and NMDS axes.

	*Cages*		*Litter Bags*	
Taxon	NMDS Axis 1	NMDS Axis 2	NMDS Axis 1	NMDS Axis 2
*Acentrella*	−0.611	−0.018	NA	NA
*Choroterpes*	−0.607	−0.343	NA	NA
Coenagrionidae	−0.456	0.160	0.492	0.310
Dytiscidae	NA	NA	−0.144	−0.322
*Erpetogomphus*	−0.356	−0.031	NA	NA
*Fallceon*	−0.539	0.061	NA	NA
*Graptocorixa*	−0.420	0.183	NA	NA
*Hyalella*	NA	NA	−0.418	−0.009
*Leptohyphes*	−0.546	−0.095	−0.214	0.571
Non-Tanypod Chironomidae	0.008	0.263	−0.475	−0.214
Physidae	NA	NA	−0.335	0.550
*Tabanus*	NA	NA	0.472	0.311
Tanypodinae	−0.407	0.668	−0.417	−0.214
Veliidae	−0.242	−0.442	−0.282	−0.188

NA indicates that the taxon represented less than 1% of all individuals collected in either cages.

**Figure 4:**
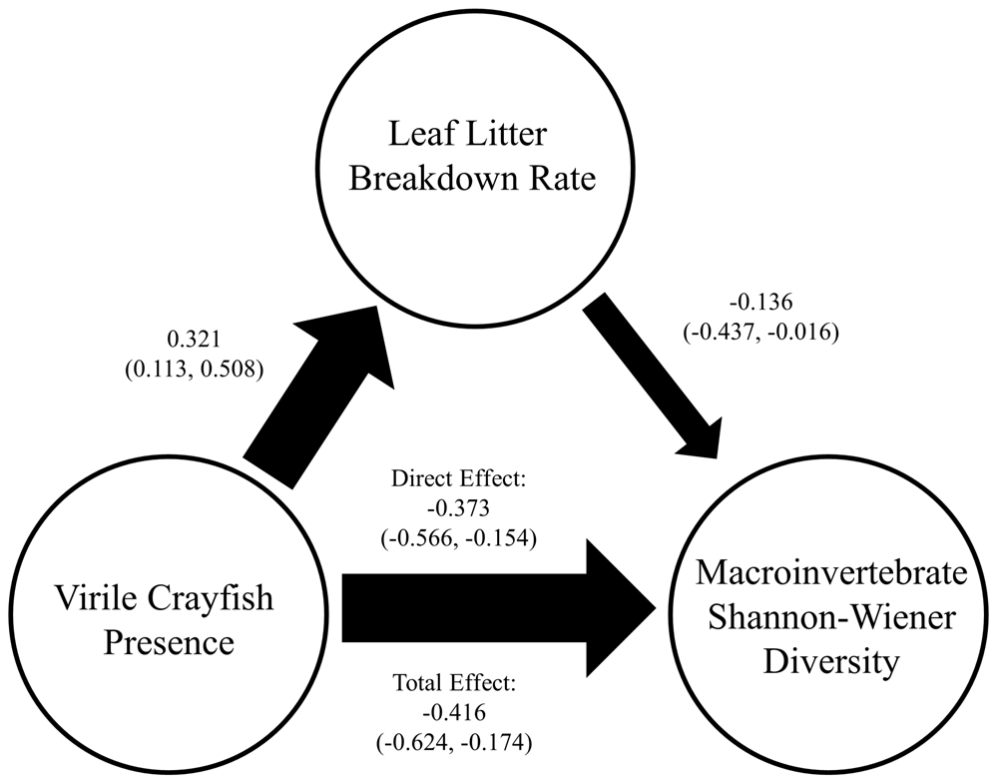
PLS path model of crayfish effects on Shannon-Wiener diversity index of macroinvertebrates colonizing leaf litter bags. Path coefficients include 95% confidence intervals in parentheses. Thickness of arrows is proportional to path coefficient for direct effect.

Virile crayfish had no significant direct or indirect effect on total macroinvertebrate abundance, i.e., the number per bag (binomial generalized linear model, *F* = 0.009, *p* = 0.924) or density, i.e., the number per g dry weight of leaf litter per bag (binomial generalized linear model, *F* = 1.188, *p* = 0.284) in leaf litter bags. By contrast, macroinvertebrate alpha diversity was directly negatively affected by virile crayfish presence (PLS path model, path coefficient = −0.373, 95% CI = −0.595, −0.156) (Figure 4). Indirect effects contributed to only 10% of the total effect on diversity, but a confidence interval could not be estimated by bootstrapping due to lack of a standard error associated with the indirect path coefficient.

## Discussion

As global change shifts species distributions, organisms with novel functional roles and/or qualities may increasingly dominate aquatic communities leading to interactions between novel species and historical communities. A primary driver of novel vegetation community establishment along desert rivers is the alteration of native flow regimes [17]. The impacts of these changes will become increasingly important as streamflow declines due to increased human water use and projected warming and drying in the Southwestern United States [5], [6], [7]. Novel consumers, such as omnivorous crayfish, may be poised to capitalize on novel resource inputs which in turn directly and indirectly affect other invertebrate consumers (Figure 4). In this experiment virile crayfish increased the breakdown rate of saltcedar leaves but did not impact breakdown of the other three native hydric and drought-tolerant species studied (Figure 2, Table 1). At the litter patch scale, i.e., the patches of benthic habitat in the stream dominated by leaf litter cover as represented by our litter bags, crayfish lowered alpha diversity of colonizing invertebrates, while the drought-tolerant leaf litter treatment significantly reduced beta diversity (Table 3). Through these combined effects, novel species in this ecosystem lowered diversity and created a more homogenous community.

### Leaf Litter Breakdown

Virile crayfish did not increase the breakdown rate of all species; only saltcedar decayed faster in the presence of crayfish (Figure 2, Table 2). These findings clearly indicate the potential of virile crayfish to use novel resources, perhaps providing a pathway for novel organic matter inputs into the food webs and biogeochemical cycles of semiarid streams. Virile crayfish are native to the upper Midwestern United States and Canada, where riparian vegetation varies but includes species of willow (*Salix*) and poplar (*Populus*) but not saltcedar (*Tamarix*) or seepwillow (*Baccharis*) [32]. Despite the fact that virile crayfish co-evolved with species closely related to native hydric species along the San Pedro River, they had the greatest impact on breakdown of saltcedar with which their native range does not overlap. Crayfish foraging decisions are based on a number of factors including food quality (i.e., C∶N ratio) and leaf toughness, thus the high food quality as indicated by low C∶N of saltcedar relative to cottonwood and willow indicates that saltcedar may be a preferred food of crayfish [24], [25], [28]. However, it is important to note that saltcedar leaves differ from the other studied species in a number of other respects that may affect crayfish foraging [23], [24], [25]; for example some crayfish are known to control the decomposition of tough recalcitrant leaves such as the drought-tolerant saltcedar in our study [44]. In order to predict how a consumer will respond to novel resources, food quality, whether defined as a stoichiometric nutrient ratio as we have done or by other metrics, must be considered as an important factor in foraging decisions as there is no clear mechanism that predicts why novel detritus will decompose differently than native detritus based on novelty alone.

Our research provides insight into differences in breakdown rate between species along this gradient of drought tolerance. In this study, seepwillow leaves decomposed faster than leaves of any other species (Figure 2). Seepwillow is not generally considered in studies of litter breakdown in arid and semiarid streams, yet it is a relatively abundant riparian plant in these systems [e.g., 23,58,59]. Additionally, our study found that saltcedar decomposed at roughly the same rate as cottonwood (Figure 2); previous studies have found that saltcedar can either decompose more slowly [58] or rapidly [59] than cottonwood in aquatic systems. These results highlight the fact that differences in breakdown rate between these two species are context-dependent. Our study supports the idea that differences between species, such as leaf food quality, can determine decomposition rates depending on other biological and physical variables that may also differ across an aridity gradient. For example, the presence of generalist consumers that feed upon novel saltcedar inputs significantly altered the rate at which saltcedar broke down in our study.

### Invertebrate Community

Virile crayfish had a significant direct impact on alpha diversity of invertebrates colonizing litter bags, but not the benthos of the cage itself (Figure 4, Table 3). These results contrast a number of studies of introduced crayfish that report effects on benthic invertebrates at the cage-level over a range of cage sizes between 0.2–6 m^2^ (mean: 1.6 m^2^) [60], indicating that crayfish effects were specifically concentrated on the litter-dwelling community in the San Pedro. In this system, as there are no specialized shredders competing for organic matter resources, crayfish likely had the greatest impact on invertebrates through direct predation or interference competition. Aside from direct predation, crayfish may also have elicited a behavioral response which inhibited colonization by certain groups or may have had indirect effects on benthic communities through alteration of habitat via consumption of plant matter and disturbance to leaf packs during feeding [31], [33], [61]. Additionally, only one of the experiments in the meta-analysis by McCarthy et al. [60] included virile crayfish, thus it is possible that this species may not affect macroinvertebrates at the same scale as other more widely-studied invasive crayfish.

Similar to crayfish effects, litter effects were only observed at the litter patch scale. We did not expect the litter treatment to affect invertebrate diversity at the cage scale because differences in food quality and habitat structure only affect the organisms that feed on and live within the litter. The drought-tolerant litter treatment was associated with a reduction in beta diversity of litter-dwelling invertebrates (Figure 3, Table 3), thus these plants may support a more homogenous community. The leaves of the four study species differ morphologically, which may provide differences in habitat complexity that affect colonization. Bailey et al. [59] reported lower invertebrate abundance and taxon richness in saltcedar than cottonwood leaf packs after three weeks in-stream and suggested morphological differences between the leaves may have played a role in structuring litter-dwelling invertebrate communities. While we found direct impacts of crayfish on litter breakdown and litter species on invertebrate richness, we surprisingly did not find an indirect effect or interaction between crayfish and litter on invertebrate diversity (Figure 4, Table 3). We expected these interactions to exist, but our study may not have had enough power to detect interactions that may have been present, or the timescale may not have been long enough for interactions to develop. However, our study was conducted over the warm, dry season when crayfish are likely to be most active in breaking down litter before it is exported in monsoonal floods [30], [44], thus we feel that the temporal scale represents the realistic period when crayfish effects should be important. It is possible that these interactions simply do not develop over this temporal scale. Additionally, the effects we found do not extend beyond leaf packs, as motile taxa that do not rely on leaf litter such as *Graptocorixa* and the mayflies *Acentrella*, *Choroterpes*, and *Fallceon* were not affected by litter composition.

### Conclusions

These results provide a sketch of the impact of the interactions between novel consumers and novel resources in semiarid stream ecosystems. However, several caveats are worth discussion. The timing of this study corresponded with the presence of YOY virile crayfish at one of our study sites, which were able to pass through cages. YOYs are primarily predatory, but a study of the signal crayfish (*Pacifastacus leniusculus*) found no effect of ontogenetic stage on crayfish impacts on leaf litter-dwelling invertebrate communities [62], [63]. YOY crayfish likely had some effect on our experimental results, but their impact is confounded with other site differences. We have treated site as a random effect to control for variation due to site differences and focus on the effects of the experimental treatments. Although virile crayfish have strong effects on detrital resources, these effects are context-dependent and may be overshadowed by direct effects of stream drying and downstream export in floods. Through effects on habitat heterogeneity and connectivity, droughts alter macroinvertebrate communities and their interactions with macroconsumers such as crayfish [14], [16]. Additionally, monsoonal flooding may export a large fraction of coarse particulate organic matter downstream [30] subsequent to the dynamics observed in this experiment. Future work should integrate flood disturbance and the effect of floods on OM budgets in this context.

Our study highlights the importance of virile crayfish to desert river organic matter dynamics. While native species do sometimes outcompete introduced competitors for novel resources [e.g., 64], there are also several examples of novel species using novel resources [e.g., 39,40]. While it is still unclear what determines native and invasive consumer use of novel resources, our research supports the hypothesis that food quality is an important factor. Omnivorous consumers are unlikely to rely on one particular resource, but abundant, high-quality invaders may subsidize their populations. Kennedy et al. [29] found that introduced red swamp crawfish abundance declined significantly after saltcedar was cleared from a desert spring, but concluded that saltcedar inputs are unlikely to facilitate exotic consumer invasion. We agree with this interpretation as *Orconectes* crayfish are omnivorous and successful invaders in a wide variety of systems [e.g., 32,36,64], but our findings support the conclusion that saltcedar invasion provides an organic matter resource that is broken down by invasive crayfish.

We found that virile crayfish had direct impacts on novel saltcedar litter breakdown and that both crayfish and litter treatments impacted the litter-associated macroinvertebrate community in the San Pedro River. Novel consumers and novel resources impacted different components of benthic invertebrate diversity, thus together these two introduced species can drastically alter community structure within detrital patches of arid streams. As surface water flow becomes increasingly variable, novel communities based on drought-tolerant litter and organisms like crayfish that consume it may also increase in abundance. Long-term studies of the entire community must be conducted to understand fully the impacts of introduced crayfish and riparian vegetation changes in semiarid streams.

## References

[1] Vitousek PM (1994) Beyond global warming–ecology and global change. Ecology 75: 1861–1876.

[2] Walther GR, Roques A, Hulme PE, Sykes MT, Pysek P, et al (2009) Alien species in a warmer world: risks and opportunities. Trends Ecol Evol 24: 686–693. 19712994 10.1016/j.tree.2009.06.008

[3] Hobbs RJ, Arico S, Aronson J, Baron JS, Bridgewater P, et al (2006) Novel ecosystems: theoretical and management aspects of the new ecological world order. Glob Ecol Biogeogr 15: 1–7.

[4] Paine RT, Tegner MJ, Johnson EA (1998) Compounded perturbations yield ecological surprises. Ecosystems 1: 535–545.

[5] Seager R, Ting M, Held I, Kushnir Y, Lu J, et al (2007) Model projections of an imminent transition to a more arid climate in Southwestern North America. Science 316: 1181–1184. 17412920 10.1126/science.1139601

[6] Serrat-Capdevila A, Valdés JB, González Pérez J, Baird K, Mata LJ, et al (2007) Modeling climate change – and uncertainty – on the hydrology of a riparian system: the San Pedro basin (Arizona/Sonora). J Hydrol 347: 48–66.

[7] Sabo JL, Sinha T, Bowling LC, Schoups GHW, Wallender WW, et al (2010) Reclaiming freshwater sustainability in the Cadillac Desert. Proc Natl Acad Sci USA 107: 21263–21270. 21149727 10.1073/pnas.1009734108 PMC3003073

[8] Stanley EH, Buschman DL, Boulton AJ, Grimm NB, Fisher SG (1994) Invertebrate resistance and resilience to intermittency in a desert stream. Am Midl Nat 131: 288–300.

[9] Sabo JL, Finlay JC, Kennedy T, Post DM (2010) The role of discharge variation in scaling of drainage area and food chain length in rivers. Science 330: 965–967. 20947729 10.1126/science.1196005

[10] Sabo JL, Post DM (2008) Quantifying periodic, stochastic, and catastrophic environmental variation. Ecol Monogr 78: 19–40.

[11] Carlisle DM, Wolock DM, Meador MR (2011) Alteration of streamflow magnitudes and potential ecological consequences: a multiregional assessment. Front Ecol Environ 9: 264–270.

[12] Lytle DA, Poff NL (2004) Adaptation to natural flow regimes. Trends Ecol Evol 19: 94–100. 16701235 10.1016/j.tree.2003.10.002

[13] Seegrist DW, Gard R (1972) Effects of floods on trout in Sagehen Creek, California. Trans Am Fish Soc 101: 478–482.

[14] Ludlam JP, Magoulick DD (2009) Spatial and temporal variation in the effects of fish and crayfish on benthic communities during stream drying. J North Am Benthol Soc 28: 371–382.

[15] Sponseller RA, Grimm NB, Boulton AJ, Sabo JL (2010) Responses of macroinvertebrate communities to long-term flow variability in a Sonoran Desert stream. Glob Chang Biol 16: 2891–2900.

[16] Bogan MT, Lytle DA (2011) Severe drought drives novel community trajectories in desert stream pools. Freshw Biol 56: 2070–2081.

[17] Stromberg JC, Bagstad KJ, Leenhouts JM, Lite SJ, Makings E (2005) Effects of stream flow intermittency on riparian vegetation of a semiarid river (San Pedro River, Arizona). River Res Appl 21: 925–938.

[18] Stromberg JC, Lite SJ, Dixon MD (2010) Effects of stream flow patterns on riparian vegetation of a semiarid river: Implications for a changing climate. River Res Appl 26: 712–729.

[19] Fisher SG, Likens GE (1973) Energy flow in Bear Brook, New Hampshire – Integrative approach to stream ecosystem metabolism. Ecol Monogr 43: 421–439.

[20] Wallace JB, Eggert SL, Meyer JL, Webster JR (1997) Multiple trophic levels of a forest stream linked to terrestrial litter inputs. Science 277: 102–104.

[21] Ball BA, Kominoski JS, Adams HE, Jones SE, Kane ES, et al (2010) Direct and terrestrial vegetation-mediated effects of environmental change on aquatic ecosystem processes. BioScience 60: 590–601.

[22] Kennedy TA, Hobbie SE (2004) Saltcedar (*Tamarix ramosissima*) invasion alters organic matter dynamics in a desert stream. Freshw Biol 49: 65–76.

[23] Schade JD, Lewis DB (2006) Plasticity in resource allocation and nitrogen-use efficiency in riparian vegetation: Implications for nitrogen retention. Ecosystems 9: 740–755.

[24] Going BM, Dudley TL (2008) Invasive riparian plant litter alters aquatic insect growth. Biol Invasions 10: 1041–1051.

[25] Moline AB, Poff NL (2008) Growth of an invertebrate shredder on native (*Populus*) and non-native (*Tamarix, Elaeagnus*) leaf litter. Freshw Biol 53: 1012–1020.

[26] Hladyz S, Gessner MO, Giller PS, Pozo J, Woodward G (2009) Resource quality and stoichiometric constraints on stream ecosystem functioning. Freshw Biol 54: 957–970.

[27] Frost PC, Stelzer RS, Lamberti GA, Elser JJ (2002) Ecological stoichiometry of trophic interactions in the benthos: understanding the role of C:N:P ratios in lentic and lotic habitats. J North Am Benthol Soc 21: 515–528.

[28] Usio N (2007) Endangered crayfish in northern Japan: distribution, abundance and microhabitat specificity in relation to stream and riparian environment. Biol Conserv 134: 517–526.

[29] Kennedy TA, Finlay JC, Hobbie SE (2005) Eradication of invasive *Tamarix ramosissima* along a desert stream increases native fish density. Ecol Appl 15: 2072–2083.

[30] Schade JD, Fisher SG (1997) Leaf litter in a Sonoran desert stream ecosystem. J North Am Benthol Soc 16: 612–626.

[31] Creed RP (1994) Direct and indirect effects of crayfish grazing in a stream community. Ecology 75: 2091–2103.

[32] Charlebois PM, Lamberti GA (1996) Invading crayfish in a Michigan stream: direct and indirect effects on periphyton and macroinvertebrates. J North Am Benthol Soc 15: 551–563.

[33] Usio N (2000) Effects of crayfish on leaf processing and invertebrate colonisation of leaves in a headwater stream: decoupling of a trophic cascade. Oecologia 124: 608–614. 28308399 10.1007/s004420000422

[34] Bobeldyk AM, Lamberti GA (2010) Stream food web responses to a large omnivorous invader, *Orconectes rusticus* (Decapoda, Cambaridae). Crustaceana 83: 641–657.

[35] Pintor LM, Soluk DA (2008) Evaluating the non-consumptive, positive effects of a predator in the persistence of an endangered species. Biol Conserv 130: 584–591.

[36] Martinez PJ (2012) Invasive crayfish in a high desert river: implications of concurrent invaders and climate change. Aquat Invasions 7: 219–234.

[37] Fisher SG, Busch DE, Grimm NB (1981) Diel feeding chronologies in two sonoran desert stream fishes, *Agosia chrysogaster* (Cyprinidae) and *Pantosteus clarki* (Catostomidae). Southwest Nat 26: 31–36.

[38] Flecker AS (1992) Fish trophic guilds and the structure of a tropical stream – weak direct vs. strong indirect effects. Ecology 73: 927–940.

[39] Helms KR, Vinson SB (2002) Widespread association of the invasive ant *Solenopsis invicta* with an invasive mealybug. Ecology 83: 2425–2438.

[40] Ermgassen PSEZ, Aldridge DC (2011) Predation by the invasive American signal crayfish, *Pacifastacus leniusculus* Dana, on the invasive zebra mussel, *Dreissena polymorpha* Pallas: the potential for control and facilitation. Hydrobiologia 658: 303–315.

[41] Turner DS, Richter HE (2011) Wet/dry mapping: Using citizen scientists to monitor the extent of perennial surface flow in dryland regions. Environ Manage 47: 497–505. 21308377 10.1007/s00267-010-9607-y PMC3056014

[42] Vandersande MW, Glenn EP, Walworth JL (2001) Tolerance of five riparian plants from the lower Colorado River to salinity drought and inundation. J Arid Environ 49: 147–159.

[43] Moody EK, Taylor CA (2012) Red swamp crawfish (*Procambarus clarkii*) discovered in the San Pedro River, Arizona: a new invader in a threatened ecosystem. Southwest Nat 57: 343–344.

[44] Huryn AD, Wallace JBW (1987) Production and litter processing by crayfish in an Appalachian mountain stream. Freshw Biol 18: 277–286.

[45] Horton JL, Kolb TE, Hart SC (2001) Responses of riparian trees to interannual variation in ground water depth in a semi-arid river basin. Plant Cell Environ 24: 293–304.

[46] Kominoski JS, Pringle CM, Ball BA, Bradford MA, Coleman DC, et al (2007) Nonadditive effects of leaf litter species diversity on breakdown dynamics in a detritus-based stream. Ecology 88: 1167–1176. 17536403 10.1890/06-0674

[47] Hauer FR, Lamberti GA (2006) Methods in stream ecology. Second Edition. San Diego, CA, Academic Press.

[48] Clarke KR, Somerfield PJ, Chapman MG (2006) On resemblance measures for ecological studies, including taxonomic dissimilarities and a zero-adjusted Bray-Curtis coefficient for denuded assemblages. J Exp Mar Biol Ecol 330: 55–80.

[49] McCune B, Grace JB (2002) Analysis of Ecological Communities. MjM Software Design, Gleneden Beach, OR. 300pp.

[50] Lennon JJ, Koleff P, Greenwood JJD, Gaston KJ (2001) The geographical structure of British bird distributions: diversity, spatial turnover, and scale. J Anim Ecol 70: 966–979.

[51] Oksanen J, Blanchet FG, Kindt R, Legendre P, Minchin PR, et al.. (2012) Vegan: community ecology package. R package version 2.0-3. http://CRAN.R-project.org/package=vegan.

[52] Henseler J, Fassott G (2010) Testing moderating effects in PLS path models: An illustration of available procedures. In: Vinzi, VE, Chinn, WW, Henseler, J, and Wang, H. (Eds.), *Handbook of Partial Least Squares*. Berlin, Germany, Springer-Verlag Berlin Heidelberg, pp. 713–735.

[53] Chao A, Shen TJ (2003) Nonparametric estimation of Shannon's index of diversity where there are unseen species in sample. Environ Ecol Stat 10: 429–443.

[54] Fox J, Weisberg S (2011) An R companion to applied regression, second edition. Thousand Oaks, CA: Sage. http://socserv.socsci.mcmaster.ca/jfox/Books/Companion.

[55] Bates D, Maechler M, Bolker B (2011) Lme4: linear mixed-effects models using S4 classes. R package version 0.999375-42. http://CRAN.R-project.org/package=lme4.

[56] Sanchez G, Trinchera L (2012) plspm: Partial Least Squares Data Analysis Methods. R package version 0.2-2. http://CRAN.R-project.org/package=plspm.

[57] Maechler M, et al.. (2011) sfsmisc: Utilities from Seminar fuer Statistik ETH Zurich. R package version 1.0-19. http://CRAN.R-project.org/package=sfsmisc.

[58] Pomeroy KE, Shannon JP, Blinn DW (2000) Leaf breakdown in a regulated desert river: Colorado River, Arizona, U.S.A. Hydrobiologia. 434: 193–199.

[59] Bailey JK, Schweitzer JA, Whitham TG (2001) Salt cedar negatively affects biodiversity of aquatic macroinvertebrates. Wetlands 21: 442–447.

[60] McCarthy JM, Hein CL, Olden JD, Vander Zanden MJ (2006) Coupling long-term studies with meta-analysis to investigate impacts of non-native crayfish on zoobenthic communities. Freshw Biol 51: 224–235.

[61] Wilson KA, Magnuson JS, Lodge DM, Hill AM, Kratz TK, et al (2004) A long-term rusty crayfish (*Orconectes rusticus*) invasion: dispersal patterns and community change in a north temperate lake. Can J Fish Aquat Sci 61: 2255–2266.

[62] Parkyn SM, Rabeni CF, Collier KJ (1997) Effects of crayfish (*Paranephrops planifrons*: *Parastacidae*) on in-stream processes and benthic faunas: a density manipulation experiment. N Z J Mar Freshw Res 31: 685–692.

[63] Bondar CA, Richardson JS (2009) Effects of ontogenetic stage and density on the ecological role of the signal crayfish (*Pacifastacus leniusculus*) in a coastal Pacific stream. J North Am Benthol Soc 28: 294–304.

[64] Olden JD, Larson ER, Mims MC (2009) Home field advantage: native signal crayfish (*Pacifastacus leniusculus*) out consume newly introduced crayfishes for invasive Chinese mystery snail (*Bellamya chinensis*). Aquat Ecol 43: 1073–1084.

